# Lung Microbiome Diversity, Infection Dynamics, and Microbe‐Mediated Cross‐Protection

**DOI:** 10.1002/mbo3.70349

**Published:** 2026-06-23

**Authors:** Sana Arooj, Akmal Zubair, Syeda Zaira Batool, Granaz Niaz, Muhammad Ali, Yasir Waheed, Eman Ramadan Elsharkawy, Naila Afghan

**Affiliations:** ^1^ Warwick Medical School Student University of Warwick Coventry UK; ^2^ Department of Biotechnology Quaid‐i‐Azam University Islamabad Islamabad Pakistan; ^3^ NUST School of Health Sciences National University of Sciences and Technology (NUST) Islamabad Pakistan; ^4^ Irfan Suat Gunsel Operational Research Center in Healthcare, Near East University Nicosia Turkey; ^5^ Széchenyi István University Győr Hungary; ^6^ VIZJA University Warsaw Poland; ^7^ Center for Health Research Northern Border University Arar Saudi Arabia; ^8^ Department of Biology Kabul University Kabul Afghanistan

**Keywords:** bacteriome, immunity, infections, lungs, microbiome, virome

## Abstract

Modern technological advances have revealed that the lungs, once believed to be sterile, actually harbor a diverse community of microorganisms. A normal lung microbiome possesses its own characteristic microbial community, although it is largely influenced by the microbiota of the upper respiratory tract. The lung microbiome is distinct from that of other organs due to unique selective pressures, including mechanical clearance through coughing, the activity of pulmonary macrophages, the coordinated movement of respiratory cilia, and the antimicrobial effects of alveolar surfactant. Although recent research has largely concentrated on the pulmonary bacteriome, comparatively little attention has been given to the lung mycobiome and virome. Various databases such as PubMed, Scopus/Web of Science, Google Scholar, and Medline for literature research up to December 2025. This updated review discusses the origin, composition, and functional significance of the lung microbiome, with particular emphasis on its protective role against respiratory pathogens through host–microbe interactions. The review primarily focuses on respiratory disorders such as asthma, along with a range of viral and bacterial infections. Special attention is given to current evidence on how lung microbial communities influence susceptibility to pulmonary infections, as well as how the lung microbiome contributes to host defense during infectious conditions.

## Graphical abstract

The protective role of the lung microbiome in maintaining respiratory health by promoting immune homeostasis, pathogen exclusion, and barrier integrity. Diverse microbial communities regulate immune maturation, enhance epithelial defenses, and contribute to antiviral responses, collectively preventing pathogen colonization and sustaining a balanced, resilient pulmonary ecosystem under physiological conditions.

## 1 Introduction

**Figure 1:**
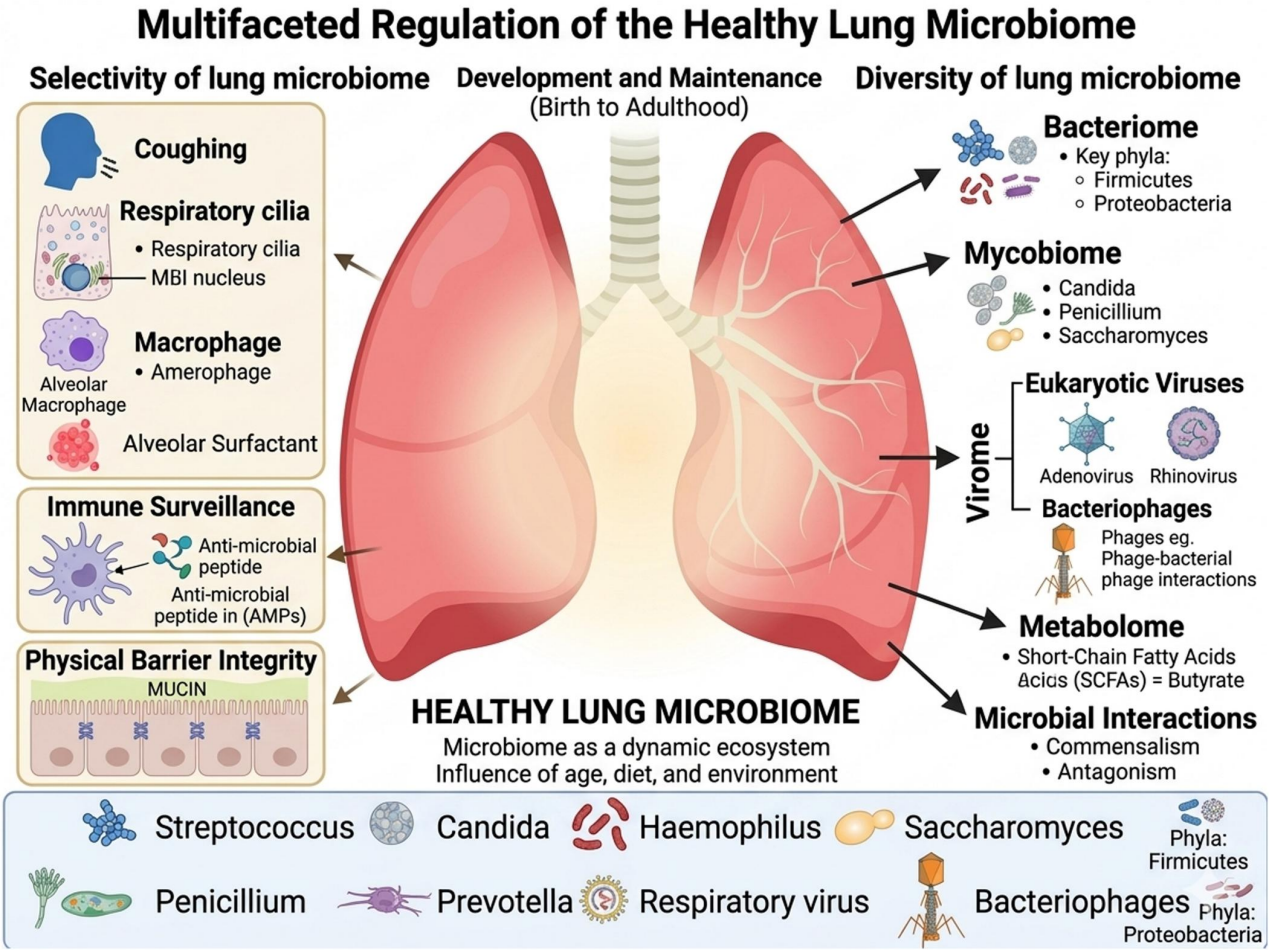
Demonstrates the complicated ecosystem of a healthy lung microbiome. It emphasizes diversity, which is subdivided into the bacteriome, mycobiome, and virome, and reveals selective processes, such as coughing and macrophage activity, that can control it. The specific composition of key microbial genera in each biome and the spatial gradients influencing colonization provide a more detailed picture of the respiratory environment.

The lungs are a paired respiratory organ located within the thoracic cavity and are enclosed by a mesothelial lining (Pandey et al. 2025). The lung is an uncommonly active organ that is sensitive to internal and external environmental changes, including exposure to other foreign particles or other hydrostatic pressure resulting from the flow of air and blood in the lungs. As the lungs have the upward degrees of elastic recoil that can be influenced by the stretches of extracellular matrix to have a more elaborate respiratory system, so lung structure and function directly influence microbiome composition (Zhao et al. 2023). The lung microbiome refers to the population of microorganisms, such as bacteria, viruses, and fungi, that inhabit the respiratory system. It plays a vital role in shaping the lung's immune responses and in defending the airways against pathogenic microbes. A stable and well‐balanced lung microbiome supports normal respiratory function, whereas microbial imbalance (dysbiosis) is associated with a higher risk of conditions such as asthma, chronic obstructive pulmonary disease (COPD), and lung cancer. The composition of the lung microbiome is strongly influenced by microorganisms from the upper respiratory tract, particularly the oral cavity, and can also be modified by factors including antibiotic use, smoking, and genetic background (Dickson et al. 2016). Recent evidence suggests that disturbances in the lung microbiome are associated with a wide spectrum of diseases, including sepsis, pneumonia, bronchiectasis, acute respiratory distress syndrome (ARDS), and lung cancer, with lung cancer being a major contributor to microbial imbalance. However, research on the lung microbiome is still in its early stages, and significant challenges remain in accurately characterizing lung anatomy and the microbial communities that inhabit it (He et al. 2017). The microbial communities consist of bacteria, viruses, and fungi (Dickson et al. 2015; Yu et al. 2016). Previously, the lungs were believed to be entirely sterile; however, recent discoveries following the Human Microbiome Project have confirmed the presence of diverse microbial communities within the lungs, each with distinct characteristics and functions in the human body. These lung microorganisms can also interact with other body microbiomes through systemic pathways such as the blood and lymphatic circulation (He et al. 2017; Johnson et al. 2019; Toma et al. 2014). The structure of the lung microbiome is shaped by the entry of microbes into the airways, their removal, and their ability to grow under local environmental conditions. In healthy lungs, microbial balance is largely maintained through an equilibrium between immigration and clearance. In contrast, during disease states, this balance is disturbed, leading to alterations in the composition of microbial communities (Yagi et al. 2021). However, the limited studies available suggest that disturbances in lung microbial communities are associated with the development and progression of lung cancer (Cullin et al. 2021). During disease states, alterations occur in both the structure and local microenvironment of the lungs, including changes in mucosal pH, oxygen levels, nutrient supply, temperature, and inflammatory conditions. These shifts favor microbial proliferation and result in modifications to the lung microbiota composition. Moreover, the lung microbiome is closely linked to immune‐related pulmonary disorders such as lung cancer, HIV, and tuberculosis, as well as complications following lung transplantation (Lee et al. 2009). According to limited research, the lung HIV microbiome project demonstrated initial lung microbiome studies in the disease area of HIV (Lozupone et al. 2013). Studies indicate that the lung microbiome plays a key role in the development of the immune system and in modulating immune tolerance during disease (Gollwitzer et al. 2014; Herbst et al. 2011). As lung microbiome disruption causes changes in entry, clearance, and local replication. However, disruption of the lung microbiome is described in most of the pulmonary diseases, but it is still unknown whether lung microbiome disruption causes inflammation or is just a consequence or marker of disease (Héry‐Arnaud et al. 2019; Martin‐Loeches et al. 2020; McGinniss et al. 2021). Further, it has been studied that the bacteriome of lungs consists of different bacterial strains such as *Proteus, Clostridium, Streptococcus, Veillonella, Pseudomonas, Haemophilus*, and *Porphyromonas* (Erb‐Downward et al. 2011; Hilty et al. 2010; Huffnagle et al. 2017). Research has shown that the lung microbial community is dynamic, varying in response to the body's immune status and the movement of microbes from the upper respiratory tract (Hilty et al. 2010; Gleeson et al. 1997). However, new findings have demonstrated that some microbial communities have components that are eventually harmful for human health, as most of the bacteria and viruses cause inflammation, which has a direct impact on the lungs (Amon and Sanderson 2017). The colonization of microbiota in lungs may not always result in a disease state, as the presence of non‐pathogenic microbes has improved the lungs’ overall health (Barko et al. 2018). Additionally, lung microbial colonization has greater diversity as compared to other tissues and organs, but it has a lower microbial population (Belizário and Faintuch 2018). The primary microbial community of the lungs generally consists of genera such as *Pseudomonas*, *Streptococcus*, *Proteus*, *Clostridium*, *Haemophilus*, *Veillonella*, and *Porphyromonas*, as shown in Figure 1 (Erb‐Downward et al. 2011).

The healthy lung microbiome has a distinct but relatively low microbial density compared with the highly diverse communities of the gut and oropharynx. Despite this lower abundance, the lung microbiome is not uniform and consists of bacterial, fungal, and viral components, collectively known as the bacteriome, mycobiome, and virome. Within the bacteriome, the most frequently detected genera include *Streptococcus*, *Veillonella*, and *Prevotella*, while *Haemophilus* species are considered characteristic resident bacteria of the lungs and are uncommon in other body sites (Li et al. 2024).

## 2 Viral Infection of the Lungs

Respiratory viral infections typically induce lung inflammation, marked by higher levels of inflammatory cytokines, including interleukin‐6 (IL‐6) and interferons (IFNs), as well as an increased presence of immune cells such as neutrophils, macrophages, and T lymphocytes (Newton et al. 2016; Tan et al. 2021). The gut plays a major role in how vulnerable people are to viral respiratory infections. These illnesses are a leading cause of death in very young children and older adults (Ruuskanen et al. 2011). Chronic lung diseases, such as COPD, asthma, and interstitial lung diseases, are major health issues because they weaken the lungs and make people more prone to viral infections. These illnesses not only make breathing harder but also highlight the need to better understand how they interact with different viruses (Britto et al. 2016). The interplay between viral pathogens and lung tissues is regulated by intricate signaling networks comprising cytokines, chemokines, and immune cells, which collectively determine the progression and outcome of the disease (Li et al. 2021). Viruses responsible for lung infections include influenza viruses, respiratory syncytial virus, rhinoviruses, and coronaviruses. These pathogens trigger a cascade of inflammatory and immune responses in the respiratory tract, which can exacerbate lung damage (Clementi et al. 2021). Lung viral infections can harm the respiratory epithelium, weakening the airway barrier and promoting bacterial colonization or secondary infections, which subsequently increase inflammation and further impair lung function (Vareille et al. 2011). Viral infections of the lungs have been recognized as an important contributor to dysbacteriosis (Li et al. 2019). New findings have demonstrated interactions between lung microbiota and respiratory tract infections (Zongjie et al. 2020). Viral lung infections can disrupt the balance between local defense mechanisms and bacterial growth by damaging the mucosal barrier, reducing bacterial clearance, promoting bacterial adherence or accumulation, and changing the composition and diversity of the lung microbiome (Mazel‐Sanchez et al. 2019). Apart from this, bacteriophages are viruses that specifically infect bacteria and establish intricate relationships with them; in the context of lung viral infections, their survival and replication are closely tied to the presence and condition of their bacterial hosts within the respiratory environment (Naureen et al. 2020). Research shows that bacteriophages, viruses that infect bacteria, play an important role in shaping which bacteria are present in the body and how abundant they are. This can sometimes disrupt the normal balance of microbes (dysbiosis) and influence how the immune system responds. Bacteriophages also serve as sources of genetic diversity by transferring virulence genes between bacterial species. Many bacterial traits involved in causing disease, such as their ability to attach, invade, or produce toxins, are controlled or carried by these phages. As a result, they can have a significant impact on respiratory health, especially during viral infections (Gogarten et al. 2002; Canchaya et al. 2003). Even though scientists understand which respiratory viruses cause disease, we still don't fully know how viruses living in different parts of the respiratory tract behave or what allows them to remain there long‐term. Research has shown that the airways of healthy children and adults contain a mix of DNA and RNA viruses, including bacteriophages, highlighting that the lungs host a complex and diverse viral community, even during infection (Willner et al. 2009; Wylie et al. 2012). Additionally, members of the recently identified DNA virus family *Anelloviridae* constitute nearly 70% of the viral population detected in the blood and various tissues of healthy individuals, suggesting their potential involvement and prevalence within the lung environment during viral infections (Delwart 2013). Studies have revealed that adults suffering from severe lower respiratory tract infections (LRTIs) display greater viral diversity within their airways. An analysis of nasopharyngeal aspirates from 210 LRTI patients identified 39 distinct viral species, predominantly belonging to the *Paramyxoviridae*, *Orthomyxoviridae*, and *Picornaviridae* families. The viruses most commonly identified included respiratory syncytial virus (RSV), metapneumovirus, *rhinovirus* types A, B, and C, along with influenza A and B viruses (Lysholm et al. 2012). While *redondoviruses* have also been found in healthy individuals, their viral genome levels are significantly elevated in critically ill patients. Importantly, the persistence of *redondoviruses* for 2–3 weeks suggests the possibility of long‐term colonization or infection in the respiratory tract (Abbas et al. 2019). Viral pathogens, including respiratory syncytial virus, primarily impact young children, leading to conditions such as rhinitis, sinusitis, or laryngitis, while neonates can develop bronchiolitis as a result of infection (Nair et al. 2010). In viral lung infections, individuals with weakened immune systems such as organ transplant recipients, cancer patients receiving chemotherapy, patients on immunosuppressive treatment for rheumatic diseases, and those with primary immunodeficiencies or HIV are especially susceptible due to impaired immune defenses (Hodinka 2016; Zubair et al. 2025a, 2025a, 2025b, 2025c; Blümel et al. 2007). Influenza A and B viruses are the primary types currently affecting humans and are major contributors to viral lung infections. They target the respiratory epithelium, eliciting immune responses that can cause inflammation, tissue injury, and reduced lung function. In severe instances, these infections can increase susceptibility to secondary bacterial infections, leading to pneumonia and other complications of the lower respiratory tract (Blümel et al. 2007). While influenza virus infections often begin with mild upper respiratory symptoms, severe cases can extend to the lower respiratory tract, resulting in viral or secondary bacterial pneumonia and significant lung damage, particularly in older adults as shown in Table 1 (Hatzifoti and Heath 2009).

**Table 1:** Major viral infections of the lungs, their cellular targets, and effect on lungs microbiome.

Virus	Primary target lung cells/Tissues	Effects on lung/Airway Microbiome	References
SARS‐CoV‐2	Ciliated epithelial cells, goblet cells, AT2 pneumocytes	Decreased diversity; increased opportunistic pathogens; gut–lung dysbiosis	Zhou et al. (2020), Wang et al. (2022)
Influenza A and B	Ciliated cells, club cells, alveolar epithelial cells	Loss of diversity; overgrowth of Streptococcus, Staphylococcus; high risk of bacterial pneumonia	Flerlage et al. (2021), Tsang et al. (2020)
RSV (Respiratory Syncytial Virus)	Ciliated bronchial/bronchiolar epithelial cells	Airway microbiome shift toward Haemophilus, Streptococcus; gut–lung axis disruption	Kristensen et al. (2024), Bergeron (2023)
Rhinovirus (RV)	Ciliated airway epithelial cells (CDHR3‐mediated entry)	Alterations in nasal/airway microbiome; increased pathobionts; exacerbation of asthma/COPD	Jackson and Gern (2022), Djeddi et al. (2024)
Human Metapneumovirus (hMPV)	Multiciliated airway epithelial cells	Reduced microbial balance; predisposition to bacterial coinfections	Heim et al. (2025), Porto (2022)
Adenovirus	Tracheal, bronchial, and bronchiolar epithelial cells; some alveolar involvement	Disturbed microbiota and increased bacterial superinfection	Lion (2014), Pichon et al. (2017)
Enterovirus D68 (EV‐D68)	Upper/lower airway epithelial cells; bronchioles	Epithelial injury facilitates bacterial overgrowth; limited direct microbiome data	Groves et al. (2018), Mattila (2024)

## 3 Bacterial Infection of Lungs

Advance findings demonstrate regarding the common belief about the respiratory system that has a composition of microbial infections of microbial infections (Prat and Lacoma 2016; D'Journo 2011; Laroumagne et al. 2013; Ioanas et al. 2002). Almost 50% of the infections in respiratory system are caused by bacterial colonization (Van Delden et al. 2020). However, the majority of deaths among patients are attributed to bacterial lung infections, most commonly pneumonia, which accounts for over 40 million cases each year and causes more than 650,000 deaths in children (Collaro et al. 2023). In children, bacterial lung infections are commonly associated with conditions such as cystic fibrosis, immune deficiencies, primary ciliary dyskinesia, and secondary lung damage following severe pneumonia (Couriel 2002). Bacterial infections of the lungs are typically marked by periods of stability that alternate with acute flare‐ups caused by various pathogenic bacteria, including *Pseudomonas aeruginosa*, *Acinetobacter baumannii*, *Klebsiella pneumoniae*, *Escherichia coli*, *Burkholderia cenocepacia*, *Achromobacter xylosoxidans*, *Staphylococcus aureus*, and species within the *Mycobacterium abscessus* complex (Pragman et al. 2016; Blanchard and Waters 2022; Delfino et al. 2015). Bacterial infections are among the most common complications following lung transplantation, representing nearly half of all infectious episodes, with pneumonia being the predominant bacterial infection affecting the lungs post‐transplant (Sims and Blumberg 2011). Pneumonia is a severe bacterial lung infection that affects populations in both developed and developing countries. It arises from bacterial‐induced inflammation of the alveoli and can be life‐threatening if not treated. The disease poses the greatest risk to vulnerable groups, including children under five and adults over sixty‐five (Baijal et al. 2014) (Piano et al. 2019; Cheruvattath and Balan 2007; Wang et al. 2016). In individuals with COPD, the lung microbiome is dominated by *Proteobacteria*, making up 51% compared to just 7% in healthy lungs, with *Haemophilus* (25%) and *Moraxella* (3%) being the most prevalent genera. Additionally, elevated levels of bacterial pathogens such as *Haemophilus influenzae*, *Moraxella catarrhalis*, *Klebsiella* spp., and *Pseudomonas aeruginosa* have been reported in these patients, contributing to bacterial lung infections (Salama et al. 2023; Ramsheh et al. 2021). Studies indicate that Proteobacteria, including *Haemophilus* and *Neisseria*, play a role in the shift from healthy lungs to a pre‐COPD state, while Bacteroidetes contribute to the progression toward COPD (Ren et al. 2026). Clinically, COPD is categorized into stable and acute exacerbation (AECOPD) phases, each with differing severity and unique lung bacterial compositions. During AECOPD, microbial diversity significantly decreases, accompanied by notable alterations in the lung microbiome. In particular, a decline in *Prevotella* and an increase in *Moraxella* have been linked to the severity of bacterial lung infections in COPD patients (Ramsheh et al. 2021; Li 2022a). Research utilizing amplicon or metagenomic sequencing indicates that bacteria such as *Granulicatella*, *Abiotrophia*, *Streptococcus*, and *Veillonella* are more common in bacterial lung infections in lung cancer patients, while *Staphylococcus* is less frequent, suggesting a protective role (Yi et al. 2023; Liu et al. 2018). Apart from this, bacterial infections remain the most common infectious complications affecting the lungs. Despite the use of advanced immunosuppressive treatments and antimicrobial prophylaxis, opportunistic bacteria can still lead to infections, with bacterial infections remaining the most common, especially within the first year after lung transplantation (Van Delden et al. 2020; Witt et al. 2012; Wojarski 2018; Nosotti et al. 2018). Additionally, respiratory conditions like bronchiectasis, which cause permanent dilation of sections of the lung airways, lead to excessive mucus buildup that encourages bacterial growth and infections, similar to cystic fibrosis. In these patients, disturbances in the normal lung microbiome heighten the risk of recurrent bacterial infections, frequently triggering acute respiratory exacerbations characterized by fever, increased sputum production, and shortness of breath (Chalmers et al. 2017). A study analyzing sputum samples from multiple bronchiectasis patients found that the bacterial phyla Firmicutes and Proteobacteria are associated with more severe forms of the disease (Lee et al. 2018). Among bacterial lung infections, *Haemophilus influenzae* is the most frequently detected pathogen, whereas *Pseudomonas aeruginosa* and *Streptococcus pneumoniae* are the leading causes of fatal cases as shown in Table 2 (Taylor et al. 2015; Ubags and Marsland 2017).

**Table 2:** Bacterial lung infections, target tissues/cell and, pathological effects.

Bacterial pathogen	Primary lung cell/Tissue targets	Key pathological effects on lung tissue/Cells	References
Klebsiella pneumonia	Airway and alveolar epithelial cells	Cytoskeletal disruption; epithelial barrier compromise; induction of cytokines/chemokines; gut–lung axis dysbiosis	Li (2022b)
Streptococcus pneumonia	Alveolar epithelial cells (type I & II), pulmonary microvascular endothelial cells, airway epithelial barrier (tight junctions/adherens junctions)	Disrupts epithelial tight junctions and adherens junctions; cytotoxicity via pneumolysin; autophagic degradation of tight‐junction proteins; immune‐cell influx (neutrophils) causing tissue damage	Xue et al. (2023)
Pseudomonas aeruginosa	Goblet and airway epithelial cells; alveolar epithelium in chronic disease	Goblet cell invasion; epithelial cell death; barrier rupture; persistent inflammation; oxidative stress; impaired tissue repair	Gustafsson et al. (2025)
Mixed/Community‐Acquired Pneumonia (various bacteria)	Bronchi, bronchioles, alveoli, lung parenchyma; immune cells (neutrophils, macrophages)	Microbiome dysbiosis (reduced richness/diversity); elevated inflammatory cytokines (IL‐6, TNF‐α); immune‐cell infiltration; partial microbiome restoration after therapy	Ye et al. (2025)

## 4 Lungs and Fungal Infections

Over recent years, the incidence of lung infections has risen, creating important diagnostic and therapeutic challenges for clinicians across the world (Jaggi et al. 2024). The new findings demonstrate that most of the fungal infections of the lungs cause 1.5 million deaths each year globally and contribute to more than a billion infections overall (Bongomin et al. 2017). With the growing population of immunocompromised individuals, fungal infections have become a significant public health concern. Opportunistic fungi such as *Aspergillus* (causing invasive aspergillosis) (Latgé 1999; Yao and Liao 2006; Carvalho et al. 2012), *Cryptococcus* (responsible for cryptococcosis) (Chayakulkeeree and Perfect 2008; Kronstad et al. 2011; Mitchell and Perfect 1995), *Pneumocystis* (leading to pneumonia) (Thomas and Limper 2004), and several region‐specific fungi are the main contributors to fungal infections in the human lungs (Lortholary 1999; Wheat 1995). Together, these organisms account for over 90% of global fungal‐related deaths (Brown et al. 2012). Current estimates show that lung‐related fungal diseases are highly prevalent, with more than 14 million cases of pulmonary aspergillosis, about 500,000 cases of *Pneumocystis jirovecii* pneumonia, and over 10 million individuals experiencing asthma aggravated by fungal sensitivity each year, in addition to significant burdens from other systemic infections such as cryptococcal meningitis and invasive candidiasis (Bongomin et al. 2017; Brown et al. 2012; Gago et al. 2019). Fungal infections of the lungs have led to a rise in invasive mycoses and related mortality, particularly among patients with severe impairments in their immune defenses (Bellocchio et al. 2004; José and Brown 2012). *Aspergillus* is among the most prevalent fungal species that produce large quantities of airborne spores (conidia), which are small, approximately 2–3 μM in size, and can readily enter the respiratory tract and alveoli. This can result in a range of lung diseases, including severe and potentially life‐threatening infections in immunocompromised individuals and exacerbations of allergic asthma (Latgé 1999; Yaguchi 2011). Pneumocystis pneumonia (PCP), caused by the fungal pathogen *Pneumocystis jirovecii*, it represents a significant fungal lung infection and is the most frequently identified AIDS‐defining illness. It can also affect non‐HIV immunocompromised individuals, especially those with weakened adaptive immunity or those undergoing prolonged, high‐dose corticosteroid treatment (José and Brown 2012). According to a study, in North America, the three primary endemic fungal infections, coccidioidomycosis, histoplasmosis, and blastomycosis, can present as community‐acquired pneumonia, making them significant contributors to fungal lung disease, as summarized in Table 3 (Hage et al. 2012).

**Table 3:** Different fungal infections of lungs, affected cells/tissues, and cellular impact.

Fungal infection	Primary lung cells/Tissues affected	Cellular and tissue‐level impact	References
Aspergillosis	Alveolar macrophages, airway epithelial cells, vascular endothelium	Conidia evade macrophage killing, germinate into hyphae; epithelial invasion causes protease‐mediated cell destruction; angioinvasion leads to thrombosis and necrosis	Kumar (2022)
Pneumocystis pneumonia (PJP)	Type II alveolar epithelial cells, alveolar macrophages, surfactant layer	Organism attaches to AT2 cells causing surfactant dysfunction; macrophage‐driven inflammation causes diffuse alveolar damage and hypoxemia	Rementeria et al. (2005)
Cryptococcosis	Alveolar macrophages, alveolar epithelium	Survives intracellularly in macrophages; capsule inhibits phagosome killing; epithelial injury enhances fungal persistence and dissemination	Apostolopoulou and Fishman (2022)
Histoplasmosis	Alveolar macrophages, lung interstitium	Yeasts survive inside macrophages by blocking phagolysosomal fusion; induces granulomatous inflammation and fibrosis	May et al. (2016)

## 5 Therapeutic Role of Bacteriome in the Lungs During Infections

**Figure 2:**
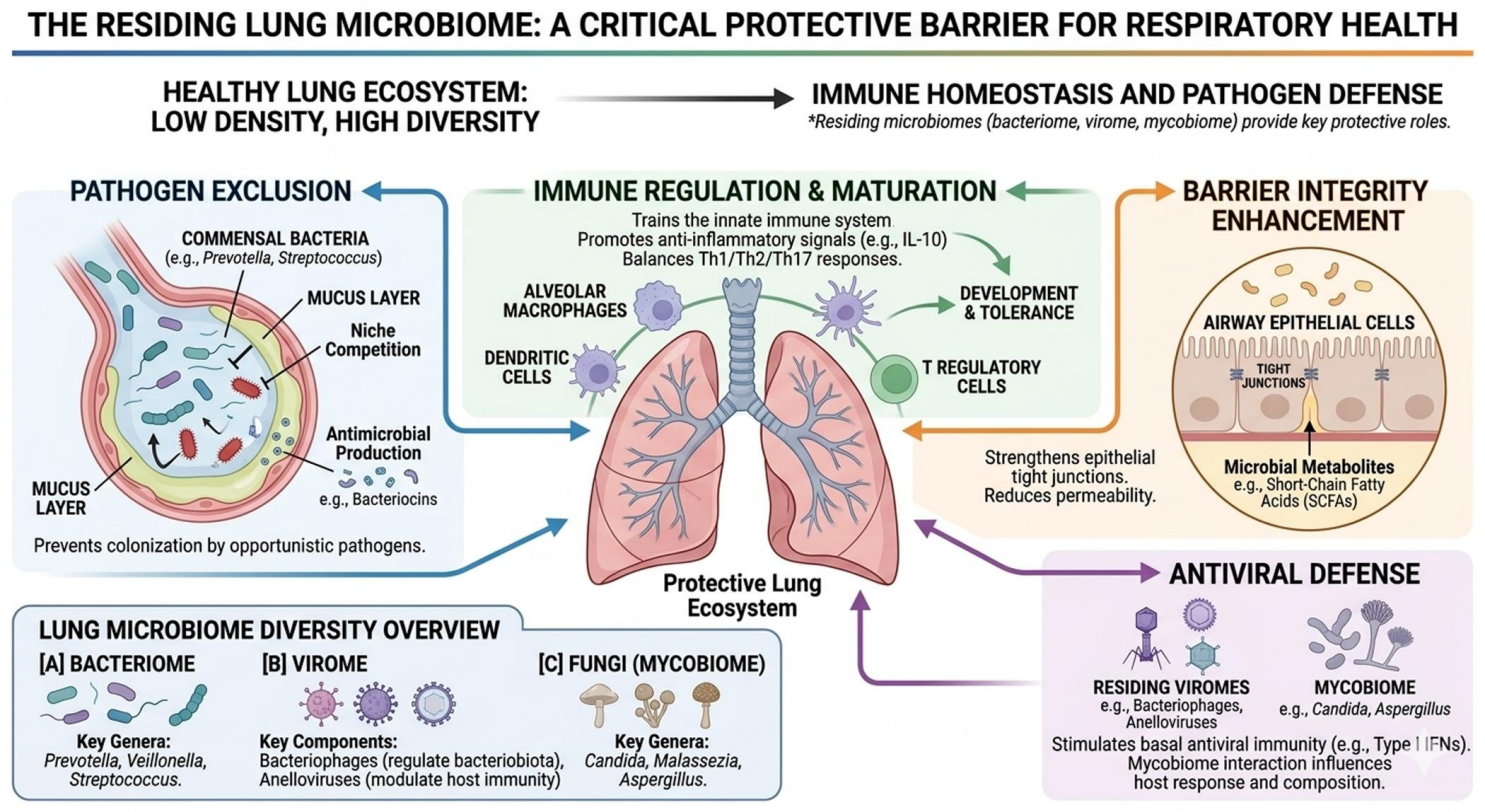
The figure shows how the bacteriome, virome, and mycobiome, the protective roles of the residing lung microbiome, in the maintenance of respiratory health. It describes the role of these commensal communities in maintaining pathogen exclusion, an increase in epithelial barrier integrity through microbial metabolites, and the maintenance of immune homeostasis. This heterogeneous ecosystem is important as it acts as a key biological barrier against infection and disease, through stimulation of basal antiviral immunity and balancing inflammatory responses.

The new findings considered the lung bacteriome as a new therapeutic target, supporting the lung's bacterial communities that prevent and treat many of the deadly respiratory infections (Dickson 2016). Recent studies have shown that bacterial communities perform distinct roles during lung infections. These investigations were conducted by amplifying the 16S rRNA genes of the bacteriome and analyzing them using next‐generation sequencing techniques (Jain et al. 2015; Kitsios 2018; Mu et al. 2021). The lung bacteriome works during infections as it interacts with the host immune system, which in response can visualize how lung infections progress and affect outcomes in both acute and chronic respiratory diseases (Man et al. 2017; Gao et al. 2014). The lung bacteriome is shaped when oral bacterial communities migrate into the lower airways, which in response influences how resident lung microbes behave during infection, and this translocation is beneficial as it introduces other extraneous microbes into the bronchoalveolar lavage fluid that become helpful in investigating further studies of the lung bacteriome during infections (Beck et al. 2015; Scher et al. 2016; Baker et al. 2021). Advances in studies of molecular and cellular pathways involved in lung infections have now visualized many infection‐related processes that shape the interactions between the host and lung bacteriome. Interest is growing in understanding how the host interacts with its lung bacteriome during infections, alongside increasing efforts to target specific bacterial species or their bioactive products, such as toxins and metabolites, as potential therapeutic strategies (Hansbro et al. 2011; Budden et al. 2017, 2019). New research shows that bacterial communities in the lungs are responsible for causing inflammation and influencing how lung infections develop, while the lung microbiome consists of different microorganisms, and it is the bacteriome in the lungs that specifically works in patterns within the airways and can directly modulate immune responses, which affects the progression and severity of lung infections (Mohajeri et al. 2018). Healthy lungs include bacterial communities by phyla such as *Bacteroidetes, Proteobacteria, Firmicutes*, and *Actinobacteria* (Faner et al. 2017). As a result of disease conditions in the lungs, this balance is disrupted and triggers different immune responses; the pathogenic species involved in this condition are *Pseudomonas aeruginosa* and *Haemophilus influenzae*. Comprehending how changes in the lung bacteriome drive the development and progression of respiratory infections is essential. In this regard, the following discussion presents recent findings on how modifications in the lung bacterial community affect infectious processes across different lung diseases as shown in Figure 2 (Shukla et al. 2017).

### 5.1 Role of Bacteriome During Asthma

Asthma is a common chronic inflammatory airway disease affecting over 330 million people worldwide, with projections estimating this number will rise to 400 million by 2025. As a result, attention has turned towards the protective behavior of the lung bacteriome, whose beneficial microbial communities may help counter airway inflammation and offer natural protective behavior against asthma. As the bacterial colonization in the lower airway is much lower than in the gut, bacterial colonies are clinically relevant and different between healthy people and people with asthma. The bacterial communities in the respiratory tract vary by anatomical site such as oropharynx, nasopharynx, sputum, Broncho alveolar lavage and as well as by age, environment and other sampling methods that has to be considered during studies (Whiteside et al. 2021). Evidence suggests that certain beneficial airway bacteria can help regulate immune responses and may protect against specific asthma subtypes. The lung bacteriome plays a critical immunoregulatory role in asthma and can impact how patients respond to therapy. Although inhaled corticosteroids remain the primary treatment for controlling airway inflammation, the composition of the airway bacterial community varies between individuals who are steroid‐naïve and those on long‐term ICS therapy. These observations suggest that the native airway bacteriome may contribute to maintaining immune balance and protecting against excessive inflammation in untreated asthma, although current evidence supporting this protective role is still limited and requires further investigation (Martin et al. 2020; Hartmann et al. 2021). Apart from this, another new finding visualizes that the bacteriome in the lungs influences how effectively asthma patients respond to corticosteroid therapy. In patients with steroid‐resistant asthma, an overgrowth of *H. parainfluenzae* has been observed. Experimental exposure of airway macrophages to this bacterium triggers stronger inflammatory activity, such as elevated IL‐8 production and p38 MAPK signaling, and reduces their sensitivity to corticosteroids. These findings suggest that a balanced airway microbiota may help maintain protective immune regulation, while dysbiosis can undermine corticosteroid effectiveness in asthma (Goleva et al. 2013). Recent findings indicate that future asthma treatments may be guided by a deeper understanding of short‐chain fatty acids, bacterial metabolites produced through the fermentation of dietary fiber. These compounds play a crucial role in linking the lung bacteriome with host immune regulation and may affect airway inflammation in asthma (Trompette et al. 2014). SCFAs are present within the airways and can be generated locally by the lung bacteriome. This highlights the protective role of bacterial metabolites in maintaining airway immune balance during asthma, as the presence of short‐chain fatty acids in the airways has been shown to strengthen immune regulation and decrease inflammation (Sulaiman et al. 2021). These findings suggest that protective members of the lung bacteriome can dampen harmful immune inflammation and help restore immune balance during asthma (Spacova et al. 2019). Another mouse study demonstrated that inhalation of aerosolized, inactivated bacteria (heat‐killed *Clostridium butyricum*) significantly suppressed allergic airway inflammation and reduced mucus production. This finding further supports the concept that components of the lung bacteriome can beneficially shape immune responses and provide protective effects in asthma (Li 2022c).

### 5.2 Role of Bacteriome During Chronic Obstructive Pulmonary Disease

COPD is a long‐term inflammatory lung condition characterized by ongoing airflow obstruction and structural damage in the distal airways (Celli and Wedzicha 2019). In COPD, disturbances in the normal lung bacteriome heighten the risk of airway infections and disease exacerbations, suggesting that a balanced bacterial community helps protect the lungs by regulating immune responses and supporting airway stability (Sethi and Murphy 2008). In people with COPD, the lung bacteriome is significantly different from that of healthy individuals, comprising a diverse microbial community rather than being dominated by a single species. This diverse bacterial ecosystem can contribute to host defense by limiting pathogen overgrowth, maintaining microbial balance, and supporting immune regulation. When this protective microbial equilibrium is disturbed, opportunistic respiratory pathogens can expand and drive inflammation and disease worsening (Sze et al. 2012). In healthy lungs, a diverse and balanced bacteriome supports epithelial integrity and maintains controlled immune surveillance through Toll‐like receptor signaling, regulatory cytokines, and IgA‐mediated mucosal defense (Sze et al. 2015). In COPD, microbial diversity is reduced and replaced by a γ‐proteobacteria‐dominant community, which disrupts this protective regulation and drives chronic inflammation (Garcia‐Nuñez et al. 2014). Changes in bacterial signals trigger innate immune pathways (such as TLRs and NF‐κB), stimulate the release of pro‐inflammatory cytokines, and increase the recruitment of CD4^+^ T cells, neutrophils, eosinophils, and B cells. *Proteobacteria* and *Actinomycetes* are closely linked to this immune cell infiltration. Therefore, although a balanced lung bacteriome normally helps regulate immune responses, its disruption in COPD drives immunity from protective functions toward chronic tissue damage and disease progression (Sze et al. 2015; Yan et al. 2022). Specific bacterial metabolites, including adenosine, 5′‐methylthioadenosine, sialic acid, tyrosine, and glutathione, are associated with improved outcomes in COPD. These molecules reflect a healthier, functionally active bacteriome that supports host defense by modulating inflammation, enhancing antioxidant capacity, and promoting tissue protection within the lungs (Segal et al. 2016; Madapoosi et al. 2022).

### 5.3 Role of Bacteriome During Cystic Fibrosis

Cystic fibrosis is caused by mutations in the CFTR gene, which impair chloride transport, resulting in thickened mucus and impaired mucociliary clearance. Although these conditions promote persistent microbial colonization and excessive immune activation, the lung bacteriome can also exert protective effects by shaping local immune responses, limiting pathogen overgrowth, and modulating chronic inflammation (Kreda et al. 2012) Conversely, a higher presence of beneficial commensal bacteria within the airway bacteriome is linked to better preservation of lung function and attenuation of inflammatory responses in cystic fibrosis, highlighting their protective influence on the diseased airway (Tony‐Odigie et al. 2022). Defective airway clearance is a central feature of cystic fibrosis, where viscous mucus retains microorganisms and limits their removal by ciliary activity and coughing. While this environment allows pathogenic bacteria to persist and form biofilms that drive chronic infection and inflammation, a balanced and protective lung bacteriome can counteract these effects by competing with pathogens, restricting biofilm development, and modulating local immune responses to reduce excessive airway inflammation (Rudkjøbing et al. 2012).

## 6 Protective Behavior of the Lungs Microbiome

The microbiome consists of all microorganisms and their genetic material, including homologous genes, present in a specific environment at a particular time (Whiteside et al. 2015). Therefore, studying the lung microbiome requires analyzing its bacterial, fungal (mycobiome), and viral (virome) components. Since the dominant populations and overall composition of the lung microbiome change dynamically with the lung's condition, various diseases can give rise to distinct microbial communities. The lung microbiome, comprising bacteria, fungi, viruses, and phages, plays a crucial role in supporting respiratory health and protecting against invasive infections (Li et al. 2024). In this section, we examine the lung microbiome, exploring its composition, functions, and protective roles during infections, with a focus on bacteria, fungi, and viruses. It highlights how these commensal microorganisms contribute to defense through three primary mechanisms.

First, beneficial microorganisms protect by competing for space and nutrients through colonization resistance, and by producing antimicrobial substances or bacteriophages that suppress harmful species. The microbiota is also present in the fetal respiratory tract during early development (Butler et al. 2019). Certain immune cells migrate to or are recruited into the lungs, where they establish themselves and shape both innate and adaptive immune responses. The lung microbiome contributes to immunity by modulating PD‐1 expression and reducing IL‐1α levels. It can affect macrophage antimicrobial function, cytokine production by immune cells, and TLR4 signaling. Additionally, the adaptive immune response within the lungs governs disease progression and, in turn, influences the ecology of the microbiome (Li et al. 2024).

Second, through immune modulation or priming, resident microbes “train” innate and adaptive immune cells, leading to faster and more appropriate responses during infection. This includes tonic interferon signaling and microbial metabolites that influence macrophage and neutrophil activity. Evidence suggests that viruses residing in the lungs play a key role in shaping and priming the host immune response. The ongoing presence of various transient viruses continuously activates antiviral immunity, potentially benefiting the host by protecting against harmful viral infections (Duerkop and Hooper 2013). Cox and his colleagues studied in a mouse model, prior exposure to a mild rhinovirus (RV) primed the respiratory tract and reduced mortality from subsequent lethal coronavirus infection, demonstrating cross‐virus priming (Cox et al. 2022).

Third, distant microorganisms and fungal communities can control lung immunity through metabolites and cytokines through cross‐kingdom and systemic interactions, like the gut–lung axis. A wider cross‐kingdom and systemic network of microbial influence is supported by new research, in addition to the local microbial communities in the lung. Distant communities, such as the gut microbiota and mycobiota, use microbial metabolites, immune‐modulatory cytokines, and even immune cell recruitment to control lung immune responses through the gut–lung axis. These defense mechanisms can be weakened, and the severity of lung infections increased if any one of these compartments, gut bacteria, gut fungi, or lung bacteria/fungi is disrupted (dysbiosis) (Sencio et al. 2021). According to Yai et al. the makeup of the gut and lung microbiota can affect both the severity of infections and the immune response. For example, more severe illnesses associated with respiratory syncytial virus (RSV) are linked to a dominance of Haemophilus influenzae and Streptococcus in the nasopharyngeal microbiome, which correlates with interferon signaling, toll‐like receptor activity, and the expression of inflammatory genes (Yagi et al. 2022). The respiratory system constantly harbors viruses and other potentially harmful microorganisms. Among the most common respiratory viruses infecting humans are influenza, rhinovirus, respiratory syncytial virus, and coronaviruses. Immune cells generate a complex response essential for clearing the virus and resolving the infection. The host microbiota has been recognized as a key factor in supporting a protective immune response against respiratory viral infections. While infections frequently upset microbial equilibrium, new research indicates that the lung microbiome also has defensive behaviors that help restrict pathogen colonization, modulate immune reactions, and maintain epithelial integrity. The microbiome serves as a biological shield, competing for nutrients, creating antimicrobial metabolites, and boosting local immunity (Bai et al. 2022).

## 7 Virome in Lungs During Infection

The term “virome” describes the viral fraction of the human microbiome, encompassing the genomes of all viruses at a specific body site, including endogenous viral elements, bacteriophages, and eukaryotic viruses that infect human cells (Porto 2022). The human virome is formed at birth, varies in abundance, variety, and composition across anatomical regions, and is temporally dynamic. Environmental factors that affect the respiratory tract virome include nutrition, location, age, mode of birth, and nursing (Purcell et al. 2025). A meta‐analysis identified over 320 viral species from 26 families in the human virome, with *Anelloviridae (anelloviruses), Papillomaviridae*, and *Bunyaviridae* being the most prevalent. Many of these viruses are “commensal” or low‐level, causing chronic or transient infections without leading to acute disease. Common phage families include *Siphoviridae, Myoviridae*, and *Podoviridae* from the order *Caudovirales*, as well as *Microviridae*. These phages are believed to help maintain a balanced bacterial community in the respiratory tract and may indirectly support the host's defense against opportunistic pathogens, as summarized in Table 4 (Wang et al. 2025).

**Table 4:** Representation of different viral/phage families present in lungs.

Category	Virus/Phage families	References
Eukaryotic viruses	*Anelloviridae, Papillomaviridae, Bunyaviridae, Picornaviridae, Orthomyxoviridae, Paramyxoviridae, Adenoviridae, Parvoviridae, Coronaviridae, Herpesviridae, Polyomaviridae*	Purcell et al. (2025)
Bacteriophages	*Siphoviridae, Myoviridae, Podoviridae, Microviridae*	Wang et al. (2025)

### 7.1 How Virome Modulate the Immune System

**Figure 3:**
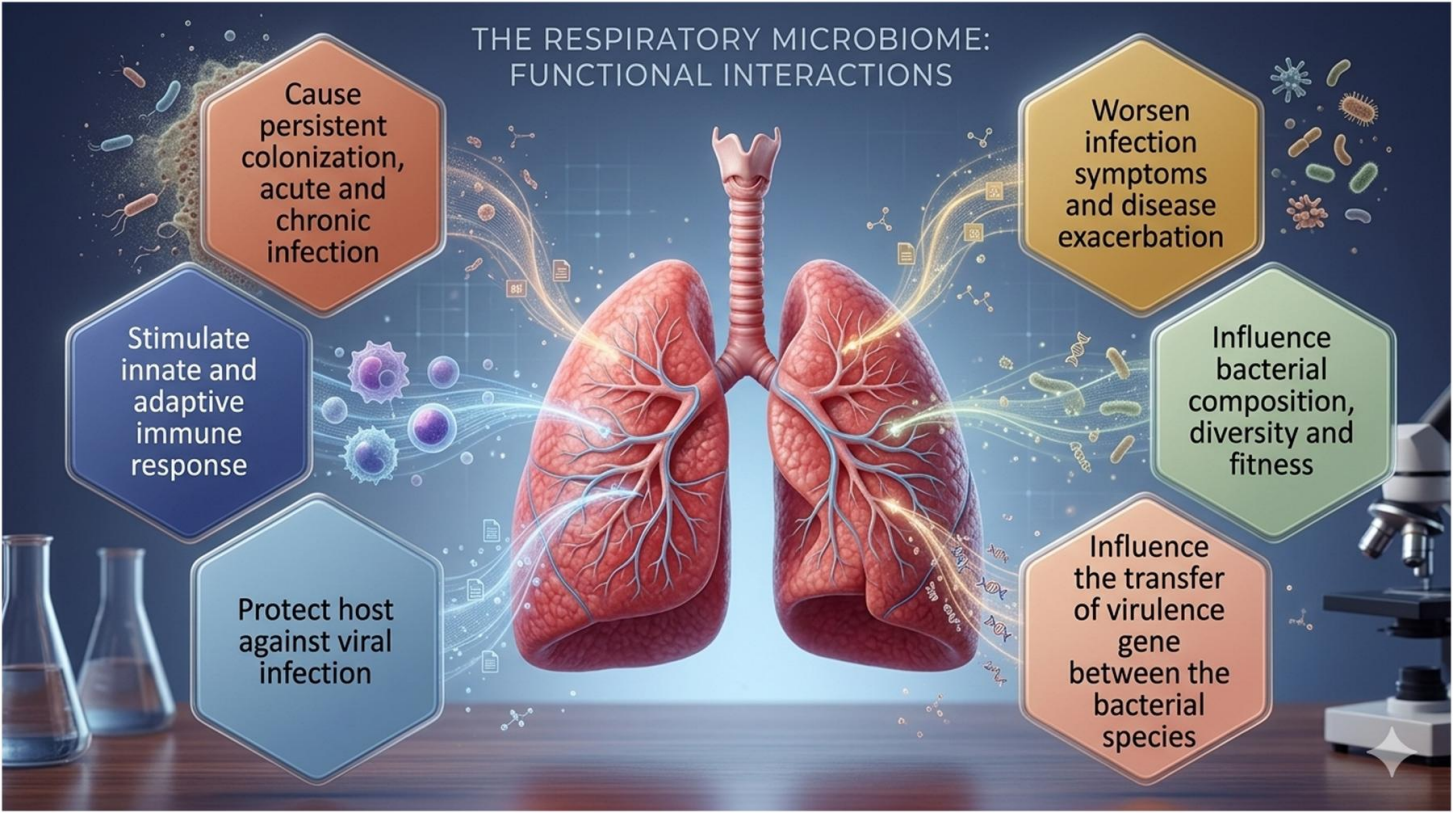
Illustration showing the dual impact of microbial interactions within the lungs during infection. The left side highlights protective roles, including stimulation of innate and adaptive immunity and prevention of viral infection.

Research has shown that the lung virome can have beneficial effects by sustaining a continuous local antiviral response. However, Porto highlights that the virome may also have detrimental effects, as higher viral loads can exacerbate disease progression whether infectious or non‐infectious and adversely impact the host (de Souza et al. 2023). The innate immune system, which keeps mucosal homeostasis and stops colonizing infectious pathogens from spreading systemically, controls the majority of early contacts between hosts and viruses. Activation of the innate immune system initiates a cascade of events that result in the release of chemokines and cytokines, often recruiting various immune cells to control pathogen invasion. Inflammasomes, which are intracellular multiprotein complexes, form in response to specific microbial, viral, or damage‐associated signals and activate downstream immune responses against these triggers. Immunity normally defends against pathogen invasion, but in those who are predisposed, reactions to innocuous antigens can result in the development of disease (Freer et al. 2018). The virome is thought to have the potential to influence the immune system, affecting the development of respiratory diseases and asthma in children. Certain viruses or viral families may impact health and disease, drive immune system maturation, and serve as potential targets for interventions by modulating immune responses. Evidence suggests that infections with common cold viruses are closely linked to the onset of respiratory and other allergic conditions. Additionally, the immune system plays a crucial role in controlling the density and composition of the microbiome to maintain homeostasis. While understanding of the respiratory virome remains limited, growing research is shedding light on the evolution of immune surveillance against eukaryotic viruses, the interactions between immune competence and non‐pathogenic viruses such as anelloviruses, and the supportive role of bacteriophages in non‐host immunity (Rovira Rubió et al. 2023). However, elevated viral loads may exacerbate the progression of both infectious and non‐infectious diseases, negatively impacting the host, as illustrated in Figure 3.

### 7.2 Changes in the Lungs Virome During Infection

Metagenomic and viromic studies show that the respiratory virome is very dynamic and changes during acute and chronic respiratory illness. In healthy airways, a relatively steady “core virome” comprising commensal eukaryotic viruses (particularly members of the *Anelloviridae*) and a varied phageome live with the host, contributing to baseline antiviral signaling and microbial homeostasis (Megremis et al. 2023). Acute respiratory infections often lead to a swift reorganization of the virome, characterized by the dominance of pathogenic eukaryotic viruses such as rhinoviruses, respiratory syncytial virus (RSV), influenza, adenoviruses, and herpesviruses that increase in abundance and richness, commensal virus representation often decreases, and bacteriophage community composition shifts in tandem with changes in the bacterial microbiota. These alterations, referred to as a “virome bloom” or “virome dysbiosis,” correspond with airway inflammation, epithelial barrier disruption, and increased susceptibility to subsequent bacterial infection (Purcell et al. 2025). Consistent patterns have been observed in various disease contexts. In pediatric pneumonia and other severe acute respiratory infections (ARIs), there is an increased detection of diverse pathogenic viruses in both the upper nasopharynx (NP) and lower bronchoalveolar lavage (sputum/BAL) samples, with distinct virome profiles differentiating affected children from healthy controls. In critically ill or immunocompromised hosts, latent DNA viruses and commensal DNA viruses (e.g., *Anelloviridae, Redondoviridae*) may increase in titer, often reflecting impaired immune control rather than direct causation, and their elevation has been associated with poor outcomes in settings such as severe COVID‐19 (Cui et al. 2024). Phage families including *Siphoviridae, Myoviridae, Podoviridae*, and *Microviridae* are typical components of the airway phageome, and their relative abundance frequently reflects colonizing bacterial taxa. Bacteriophage (phage) dynamics follow changes in the bacterial population. Phage populations can become disrupted during infections or chronic conditions like cystic fibrosis and bronchiectasis. This instability may result from phage blooms targeting dominant pathogens such as Pseudomonas, Staphylococcus, and Streptococcus, or from reduced phage diversity, which can promote horizontal gene transfer, the dissemination of antibiotic resistance genes, and alterations in bacterial virulence (Zamora et al. 2024). Overall, the research points to a model where a healthy virome helps maintain immunological homeostasis, while infection‐associated virome disruption, such as the growth of pathogenic viruses, changes in commensal DNA viruses, and rearrangements of phage‐bacteria, increases inflammation and can lead to secondary infections or the advancement of disease. Standardized, long‐term, multi‐omic research integrating transcriptome data from bacteria, fungi, viruses, and hosts will be necessary to fill in the gaps and determine therapeutic or biomarker correlations, as shown in Table 5 (Abd El‐Aziz et al. 2019).

**Table 5:** Summaries the selected respiratory virome studies key findings and limitations.

Sr. no	Name of author	Year	Host	Infection type	Finding	limitations	References
1	Waters et al.	2017	Murine inhalation models; case reports	Preclinical/chronic *P. aeruginosa* lung infection models	Phage therapy improves outcomes in chronic lung disease models and reduces the burden of P. aeruginosa.	Preclinical and compassionate use data; controlled human trials limited.	Waters et al. (2017)
2	Abd El‐Aziz et al.	2019	Mouse — acute lung infection model	Acute *P. aeruginosa* lung infection (mouse)	Phage therapy improved bacterial elimination and complement‐mediated lysis, functioning synergistically with host immunity.	Animal model; single phage strain tested.	Abd El‐Aziz et al. (2019)
3	Sweere JM (Sweere et al.)	2019	Murine and human immune cells/wound models	Mechanistic (phage‐immune interactions)	Antiviral signaling via TLR3/TRIF is activated by filamentous Pf phages, potentially obstructing microbial elimination (mechanistic link).	Model systems; bacterial wound infections rather than lung in vivo.	Sweere et al. (2019)
4	Merenstein C (Merenstein et al.)	2021	Adults — oropharyngeal/respiratory samples	COVID‐19 (hospitalized)	In severe instances, Anelloviridae and Redondoviridae are enriched; virome characteristics are linked to severity.	Correlative; heterogenous sampling.	Merenstein et al. (2021)
6	Megremis S (Megremis et al.)	2023	Children — nasopharyngeal samples	Preschool asthma/stable vs recurrent RTI	There were changes in virome between asthmatic and healthy preschoolers, with recurrent RTI having a higher harmful viral richness	Cross‐sectional; regional cohort.	Megremis et al. (2023)
7	Cao L (Cao et al.)	2023	Children — URT longitudinal swabs	Longitudinal anellome dynamics (ARTI)	ARTI episodes are related to the persistent, unique “anellome” that each kid carried.	Focus on anelloviruses; limited geographic diversity.	Cao et al. (2023)
8	(Zamora et al.)	2024	Human respiratory epithelial cells	In vitro epithelial response to lytic phages	Lytic phages directly modulate the host by causing airway epithelial cells to secrete proinflammatory and antiviral cytokines.	In vitro; requires in vivo confirmation.	Zamora et al. (2024)
9	Eiselt VA (Eiselt et al.)	2024	Review (preclinical + clinical reports)	Literature review — phage therapy for MDR *P. aeruginosa*	summarizes the data in favor of phage treatment for MDR pulmonary Pseudomonas infections and emphasizes the immune system's and antibiotics’ cooperation.	Heterogeneity of included studies; need for RCTs.	Eiselt et al. (2024)

### 7.3 Bacteriophage Mediated Protection

Bacteriophages are viruses that infect bacteria, composed of infectious particles containing at least protein and nucleic acid. In the human gastrointestinal tract, phage populations are often dominated by Caudovirales, including *Myoviridae, Podoviridae, Siphoviridae*, and *Microviridae*, while the respiratory tract and oral cavity also commonly host Caudovirales (Liang and Bushman 2021). The majority of viruses in a healthy lung are known as phageomes, which frame the bacterial population and may be crucial to lung health and immunity (Jankauskaitė et al. 2018). Bacteriophages have also been found to influence the eukaryotic microbiome and play a significant role in regulating the mammalian immune system (Federici et al. 2021). Bacteriophages can influence both health and disease by modifying their bacterial hosts and interacting with the immune system. Numerous studies have shown that phages act as reservoirs of virulence genes, as they encode a wide range of bacterial virulence factors that regulate bacterial fitness, colonization, adhesion, invasion, and toxin production. In addition, bacteriophages contribute to the maintenance and spread of antibiotic resistance genes (Dodi et al. 2021). Bacteriophages are thought to play a major role in shaping the composition and abundance of the bacterial microbiota, contributing to dysbiosis and potentially altering the host immune response. By facilitating the transfer of virulence genes between bacterial species, bacteriophages act as reservoirs of genetic diversity within microbial communities. Many bacterial virulence traits, including those involved in colonization, adhesion, invasion, and toxin production, are phage‐encoded. Consequently, the acquisition of these genes by bacteria is likely to influence host immunity as well as the diversity and functional behavior of the microbiota. Through their interactions with bacteria, phages can have both direct and indirect interactions with host cells. Dendritic cells have been shown to vigorously phagocytose phage particles in the past (Porto 2022).

There are few mechanism through which phages provide protection during infection.

#### 7.3.1 Targeting Bacterial Pathogens

Phages can specifically target bacterial pathogens in the lung environment, offering a promising therapeutic alternative where conventional antibiotics fail. A comprehensive review found that phages directed against *Pseudomonas aeruginosa* in pulmonary infections including multidrug‐resistant (MDR) strains were able to lyse bacteria and significantly re duce disease severity in both animal models and human cases (Eiselt et al. 2024). These findings emphasize the promise of phage therapy as an effective antimicrobial strategy for managing hard‐to‐treat respiratory infections, such as those associated with cystic fibrosis or ventilator‐associated pneumonia. In addition to their antibacterial activity, phages also interact with the host immune system. Sweere et al. showed that when dendritic cells engulf *Pseudomonas aeruginosa*. This indicates that bacteriophages can modulate the immune microenvironment by inducing antiviral signaling cascades, which may influence both bacterial persistence and inflammatory outcomes in the lung. Such immune interactions suggest that phages contribute not only to direct bacterial control but also to the broader immunological landscape that shapes infection dynamics (Sweere et al. 2019).

#### 7.3.2 Disrupting Biofilms

Another key function of bacteriophages in lung infections is their ability to penetrate and disrupt bacterial biofilms, which are frequently present in chronic lung conditions such as cystic fibrosis and bronchiectasis, shelter bacteria from medications and host defense responses, resulting in persistent infections. Phages have particular enzymes, such as depolymerases, that may break down the extracellular matrix of biofilms, allowing them to reach and lyse the bacteria contained inside. Waters et al. reported that phage therapy was highly effective in reducing *P. aeruginosa* biofilms in chronic lung infection models, resulting in decreased bacterial load, alleviated inflammation, and improved lung function. This biofilm‐targeting capacity is a major advantage of phages over antibiotics, as biofilm‐associated bacteria are typically up to a thousand times more resistant to chemical treatment. Therefore, by dismantling biofilms and destroying bacterial colonies, phages help restore microbial balance in the lungs and facilitate more effective immune clearance (Waters et al. 2017).

#### 7.3.3 Synergy With the Immune System (and Immunomodulatory Effects)

Phage host immune interactions are complex and bidirectional: phages can synergize with innate immune mechanisms to enhance bacterial clearance, but they can also engage antiviral sensing pathways that paradoxically impair bacterial clearance under some conditions. On the synergistic side, several studies indicate that phage therapy enhances host bactericidal mechanisms. For instance, phage therapy has been shown to enhance complement‐driven killing of serum‐resistant *Pseudomonas aeruginosa* strains and to speed up bacterial clearance in acute lung infection models, indicating that cooperation between phages and the complement system is a key mechanism underlying improved outcomes. Abd El‐Aziz et al. also reported increased complement‐mediated bacterial lysis and more efficient clearance following phage treatment in a mouse model of acute *P. aeruginosa* lung infection, supporting the idea that phages can work synergistically with humoral components of the innate immune system (Abd El‐Aziz et al. 2019).

Phages are also capable of influencing cellular immune responses, as they can be internalized by innate immune cells such as dendritic cells and macrophages, as well as by epithelial cells, and their nucleic acids or structural proteins can be sensed by pattern recognition receptors. Sweere et al. described a notable example in which uptake of a specific filamentous phage (Pf) by immune cells triggered TLR3/TRIF‐dependent type I interferon signaling, which suppressed phagocytic clearance and thereby impeded bacterial elimination in certain context an effect that links phage presence to maladaptive antiviral‐like responses during bacterial infection. Similarly, more recent work of Zamora et al. shows that lytic phages stimulate respiratory epithelial cells to secrete antiviral and proinflammatory cytokines (including IFN‐β, IL‐6, TNF‐α), demonstrating direct epithelial sensing of phages and potential downstream effects on inflammation and immune cell recruitment (Zamora et al. 2024).

### 7.4 Eukaryotic Virus‐Mediated Protection

The respiratory virome contains a number of taxa of eukaryotic viruses that survive in healthy people in a nonpathogenic condition. *Anelloviridae* are eukaryotic viruses commonly detected in healthy airways and are considered potential commensals; however, they have also been linked to disease conditions and states of immunosuppression (Young et al. 2015). The nasopharynx of healthy children has been found to harbor several pathogenic viral families, including *Picornaviridae (human rhinovirus), Adenoviridae, Coronaviridae, Orthomyxoviridae (*influenza viruses), *Paramyxoviridae* (respiratory syncytial virus), *and Parvoviridae* (human bocavirus) (Ogunbayo et al. 2022). The nasal virome of both healthy adults and children commonly harbors DNA viruses, including members of the *Papillomaviridae, Polyomaviridae, Herpesviridae*, and *Adenoviridae* families. Their existence might indicate brief exposure or asymptomatic transport, with the innate immune system mediating their removal (Sandybayev et al. 2024). Some eukaryotic viruses, such as those belonging to the *Anelloviridae* family, may be commensals rather than traditional pathogens of the human respiratory system, according to more recent findings (Cao et al. 2023; Kaczorowska and van der Hoek 2020). Anelloviridae has been detected in both healthy individuals and patients with acute respiratory infections (ARI) or pneumonia, although it has not been demonstrated to be a direct cause of the illness. This supports the theory that these viruses may not be obvious pathogens but rather innocuous inhabitants or immune modulators (Megremis et al. 2023).

**Figure 4:**
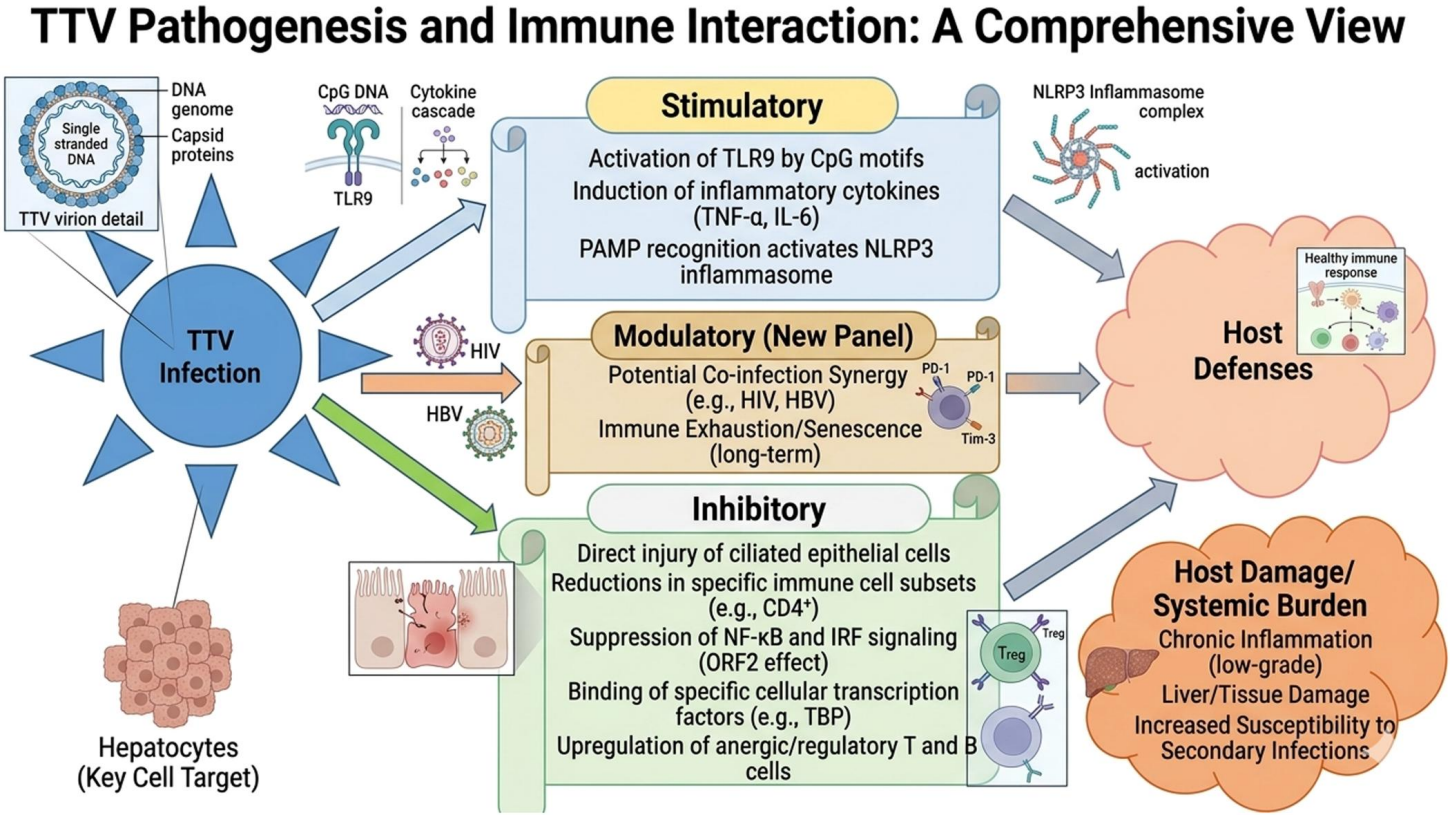
Illustrates the dual Stimulatory and Inhibitory mechanisms by which Torque Teno Virus (TTV) infection interacts with Host Defenses. The Stimulatory pathway includes the activation of TLR9 and PAMP recognition, leading to inflammasome activation, while the Inhibitory pathway involves suppression of immune cells (e.g., NF‐kB, T and B cells) and injury to ciliated cells, ultimately suppressing the host's ability to mount a defense.

Notably, research indicates that anelloviruses may affect the immune system, which in turn can shape the microbial environment within the lungs. For example, it was discovered that TTV DNA load was linked to immunological indicators of activation and was proposed as a biomarker of immune competence. In order to prevent the proliferation of bacterial pathogens or the severe usage of viral co‐infection, a moderate viral concentration may help maintain the immunological vigilance state at balanced immune activation (Freer et al. 2018). An elevated Torque teno virus (TTV) load, however, may signal dysbiosis or immune exhaustion, particularly during immune suppression or chronic inflammatory conditions such as asthma, chronic obstructive pulmonary disease (COPD), or bronchiectasis. In these situations, airflow restriction and illness aggravation have been linked to TTV abundance (Merenstein et al. 2021). The idea that these non‐pathogenic viruses may function as ecosystem modulators within the respiratory tract is further supported by the discovery of another commensal viral family, Redondoviridae, which also exhibits high abundance in critically ill individuals and correlates with altered bacterial community structure. Figure 4 illustrates the dual Stimulatory and Inhibitory mechanisms by which Torque Teno Virus (TTV) infection interacts with Host Defenses (Megremis et al. 2023).

## 8 Mycobiome in Lungs During Infection

The commensal fungi that live in the respiratory system are referred to as the “lung mycobiome” (Katsoulis et al. 2024). The makeup of the mycobiome in the respiratory tract is continuously shifting from early colonization during birth through adolescence and old life. Various factors, both vertical (from the mother) and horizontal (from the environment), shape the diversity of fungal species in the airways. These factors include the mode of delivery, gestational age, and feeding practices, while diet, body weight, and geographic location remain important influences throughout childhood into adulthood (Strati 2016). The ability of transient fungal communities to establish long‐term colonization in the airways depends on the specific species, their microbial load, and the condition of the host's lungs (Huffnagle et al. 2017). Although there is often overlap in ecosystem composition, the respiratory mycobiome can be divided into two connected regions: the upper and lower respiratory tracts (URT and LRT). Previously, fungal colonization of the respiratory system was thought to be temporary, as early studies focused mainly on potentially pathogenic fungi. However, more recent research has revealed that fungi can establish permanent residence in the respiratory tract, with certain genera and species showing a preference for colonizing the airways rather than the oral cavity (Belvoncikova et al. 2022). *Cladosporium, Eurotium*, and *Aspergilus* are the most often encountered genera in lung tissue, and the majority of fungi found in the human respiratory system are members of the phyla Basidiomycota and Ascomycota. Many genera and species make up the lung mycobiome of healthy people, however external factors like Aspergillus species predominate. Similarly, environmental molds like *Cladosporium cladosporioides* and *Eremotheciumsinecaudum*, which were usually isolated from water, plant, or specimens of soil, were the most prevalent species identified by Woerden et al (Nguyen et al. 2015). Structural ligands and metabolites produced by the lung microbiota, including bacteria, viruses, and fungi, exert diverse effects on both innate and adaptive immunity, highlighting their role in biologically regulating immune development and function (Jiang et al. 2017). *Candida albicans, Candida fumigatus*, and other fungi may transition from commensalism to pathogenicity as a result of immunodeficiency, disturbance of the normal microbiota, and compromised host barrier functions. These opportunistic fungal illnesses have been shown to have both beneficial and detrimental effects on the human immune system. Pre‐exposure to *Candida albicans* primed monocytes for a pro‐inflammatory phenotype, which led to enhanced TNF‐α and IL‐6 production following activation with TLR ligands. Administration of β‐1,3‐glucan alone, which is detected by the receptor dectin‐1, may mimic this effect. However, it remains unclear whether resident *C. albicans* in the mycobiota exerts the same immune “training” effect in vivo, or how this is balanced with the need to suppress excessive immune responses and maintain tolerance toward commensal microbiota (Quintin et al. 2012). Fungi modulate the host immune system in order to reduce immune responses. For example, it has been shown that *Candida albicans* specifically activates the TLR2 receptor, resulting in a Th2 immune response that allows it to persist in the absence of a strong pro‐inflammatory Th1 response. It also generates farnesol, which significantly reduces the cytokine production of macrophages. When *A. fumigatus* converts tryptophan to kynurenine via the host enzyme indolamine 2,3‐dioxygenase, immunodeficiency results (Krupa and Kowalska 2021).

## 9 Changes in the Lungs Mycobiome During Infection

The lung mycobiome is often disturbed by infection and inflammation. Acute infections and chronic respiratory conditions can cause fungal populations to deviate from a “healthy” baseline. For instance, *Aspergillus* species growth in sputum is linked to exacerbations of chronic obstructive pulmonary disease (COPD), while *Aspergillus fumigatus* and *Candida* often colonize airways during pulmonary infections in people with cystic fibrosis (CF) (Garaci et al. 2024). Fungal overgrowth is often seen in bronchial lavage and endotracheal samples from ICU patients with acute viral pneumonia, such as severe COVID‐19. According to studies, COVID‐19 lungs exhibit blooming of oral bacteria together with enrichment of Candida and even environmental molds like Rhizopus or Mucor; these mixed communities are correlated with elevated indicators of inflammation. All things considered, lung infections caused by bacteria or viruses may cause mycobiome dysbiosis, in which opportunistic fungus proliferate when the natural community balance is upset. The precise patterns vary depending on the disease agent: bacterial pneumonia may produce niches for fungus by changing pH, oxygen, and antimicrobial pressure, whereas viral infections often permit poly‐microbial expansion (viruses may disrupt epithelial barriers) (Merenstein et al. 2022).

## 10 Protective Roles of the Lungs Mycobiome

According to new research, commensal lung fungus may shield the host against illness. One such example is the conserved fungal cell‐wall polysaccharide β‐glucan. Mice given β‐1,3‐glucan prior to infection had significantly increased resistance to pulmonary infections. Pretreatment with β‐glucan increased survival in influenza A virus models: treated mice had less weight loss, less lung inflammation, and specialized IL‐10‐releasing neutrophils (also known as regulatory neutrophils) in the lung (Khan et al. 2025). By preventing tissue damage during a flu infection, these neutrophils showed that they have a fungal‐driven disease tolerance strategy. In a similar vein, mice that received β‐glucan before being challenged by Mycobacterium TB exhibited “trained immunity,” as their bone marrow myeloid progenitors grew and generated extremely active phagocytes. IL‐1 signaling was necessary for this β‐glucan training, which significantly enhanced lung growth in bacterial regulation (Kaufmann et al. 2018). Therefore, by reprogramming innate immunity, fungal elements may provide cross‐protection against bacterial infection. Additional inter‐kingdom effects have been observed: commensal Candida albicans colonization in the stomach increases Th1 cells that cross‐react with Aspergillus antigens, possibly shielding the lung against intrusive mold infection (via a gut–lung axis, in mice) (Sey and Warris 2024). These animal studies suggest that a healthy lung mycobiome or its molecular patterns may prepare host defenses widely, notwithstanding the paucity of human evidence. Furthermore, some commensal fungi or their byproducts may directly prevent the development of infections (e.g., *Pichia yeast* may release substances that impede the growth of *Aspergillus* or *Candida* in vitro, indicating a competitive function in polymicrobial environments) (Katsoulis et al. 2024). Both immunity control and microbe–microbe interactions seem to be involved in the protective benefits of the mycobiome. Pattern‐recognition receptors that identify fungi are important components of the immune system. For example, the C‐type lectin receptor Dectin‐1 recognizes β‐glucan and helps regulate inflammation by promoting the phagocytosis of inhaled fungal conidia and stimulating the production of the anti‐inflammatory cytokine IL‐10. Acting as the first line of defense, alveolar macrophages, along with recruited neutrophils, then destroy the fungal spores through oxidative bursts (Patin et al. 2019). Additionally, adaptive immunity is shaped: Th1 cells (which generate IFNγ/TNF) improve fungus clearance, but Th2‐skewed actions may exacerbate lung allergies (Sales‐Campos et al. 2013). β‐glucan reprograms bone marrow haematopoietic stem cells to produce more neutrophils and monocytes in trained immunity systems. Previous fungal exposure increases resistance to unrelated diseases because these “developed” cells are more receptive to new threats (Kaufmann et al. 2018). A further layer is provided by interactions between bacteria. In the airway, bacteria and fungi often combine to produce mixed biofilms or communities. Fungal–bacterial biofilms may shield microorganisms from the host, according to in vitro and animal research. For instance, a mixed *Candida–Pseudomonas* biofilm is more resistant to immune clearance than either alone (Zhang et al. 2022). Additionally, there is chemical crosstalk: fungal metabolites, such as Aspergillus gliotoxin, may suppress bacterial neighbors, while bacterial products, such as signaling peptides or short‐chain fatty acids, can restrict fungal growth or pathogenicity. These cross‐kingdom impacts may alter infection progress. For example, *A. fumigatus* generates phenazines that decrease *P. aeruginosa* biofilm development, whereas Pseudomonas aeruginosa secretes rhamnolipids that inhibit *A. fumigatus* growth. These interactions show how the mycobiome may indirectly protect the host by limiting co‐infecting pathogens, despite the fact that they are often investigated in illness circumstances (Peleg et al. 2010). All things considered, lung fungi work with bacteria and viruses directly to enhance host defense by interacting with PRRs and adaptive mechanisms.

## 11 Differences Across Infection Types

The preventative influence of the mycobiome differs according to the kind of illness. In bacterial lung infections (e.g., TB, pneumonia), fungal signals mostly enhance host resistance. β‐glucan‐activated macrophages and neutrophils exhibit enhanced bacterial lethality (Medzhitov et al. 2012). With contrast, with viral illnesses such as influenza, the focus is often on mitigating immunopathology. β‐glucan initiated a disease tolerance program in neutrophils, resulting in the production of IL‐10, which safeguarded lung tissue without directly influencing virus load. In this context, the mycobiome aids in regulating the immune response during viral infections, hence avoiding “cytokine storms” rather than enhancing cytotoxicity. In the context of fungal challenges, such as exposure to Aspergillus, the situation is complex: previous fungal colonization may enhance antifungal Th1/Th17 responses and promote clearance, while an overactive Th17 response might lead to allergy harm (Khan et al. 2025). Protective immunity against inhaled molds need robust Th1 and neutrophil activity, whereas Th2 or unregulated Th17 responses may aggravate symptoms. Consequently, commensal fungi may safeguard against subsequent fungal infections by instructing the immune system, but the precise result is contingent upon the equilibrium of T cell responses. In conclusion, fungal commensals seem to bias host defense mechanisms to address pathogen‐specific requirements: augmenting microbicidal activity in bacterial infections, activating regulatory pathways in viral infections, and establishing antifungal immunity in fungal infections (Sales‐Campos et al. 2013). This Table 6 summarizes the lung fungal communities, their alterations during infection, and their immunomodulatory functions that are described in peer‐reviewed papers and reviews (2020–2025).

**Table 6:** Representations of fungal community alternation during lung infections.

Sr. no	Name of author	Year	Host	Infection type	Finding	References
1	Khan et al.	2025	Mouse	Influenza A (viral)	Pre‐treatment with β‐Glucan enhanced IL‐10^+ regulatory neutrophils and lung tissue tolerance, resulting in enhanced survival ratios.	Khan et al. (2025)
2	Moorlag et al.	2020	Mouse	*Mycobacterium tuberculosis* (bacterial)	β‐glucan enhanced host resistance to TB by eliciting trained immunity in monocytes and haematopoietic stem cells via IL‐1.	Moorlag et al. (2020)
3	Katsoulis et al.	2024	Mouse	Chronic lung disease	Analysis of airway mycobiome in healthy and sick lungs; highlights immunological linkages and dysbiosis in COPD and CF.	Katsoulis et al. (2024)
4	Garaci et al.	2024	Human	Respiratory infections	Review of lung bacteriome‐mycobiome interplay; addresses immunological and metabolic processes of microbial interactions	Garaci et al. (2024)
5	Bernardes et al.	2020	Human	Asthma/Allergy (virus)	An examination of gut and lung mycobiomes in asthma suggests that early‐life fungal contact contributes to atopy and inflammation of the airway.	van Tilburg Bernardes et al. (2020)

## 12 Conclusion and Future Direction

Leukocytes, mycobacteria, and viruses are key components of the lung microbiome. The majority of this microbiome is bacterial, such as Ralstonia originating from the upper respiratory tract, similar to the microbial communities found in the oropharynx. Bacteria located in the lungs, such as *Haemophilus*, are absent from the oral cavity. The lung microbiome plays a key role in regulating the pulmonary environment and immune responses, helping maintain lung homeostasis. Unlike a static community, it is dynamic and continuously evolving. There is bidirectional regulation between the lung microbiome and the microbiomes of the gut and oral cavity. Changes in the oropharyngeal microbiome can influence the lungs, and vice versa, through the gut‐lung axis, which highlights the metabolic and immunological connections between these organs. Metabolites such as short‐chain fatty acids (SCFAs) produced by the gut microbiome can modulate lung immune responses, while alterations in the lung microbiome may affect gastrointestinal health. However, research on the gut‐lung axis is still in its early stages, and the roles of genes and metabolites in lung microbiota remain largely unexplored [202–204]. Decades of studies have linked the lung microbiome to various respiratory diseases, including asthma, chronic obstructive pulmonary disease (COPD), cystic fibrosis (CF), and the recent COVID‐19 pandemic. Disease states often alter the abundance and composition of key bacterial genera, indicating that disruptions in lung microbiota can contribute to disease onset and progression. Recent studies also suggest a connection between the lung microbiome and lung cancer. Dysbiosis or chronic inflammation driven by microbial metabolites may induce cellular DNA damage, promoting cancer development. High microbial loads during cancer treatment can reduce immunotherapy efficacy, and a heavy bacterial presence is associated with poorer prognosis. The microbiome composition of healthy individuals differs markedly from that of lung cancer patients, suggesting that specific microbial components or bacterial toxins could serve as biomarkers for diagnosis and treatment planning.

Future research will explore additional factors potentially associated with the lung microbiota. Both the presence of bacteria in the lungs and their metabolites could serve as diagnostic markers for disease progression. Respiratory disorders such as asthma and COPD are linked to distinct microbial communities, and quantitative shifts in the microbiota may allow for early disease detection and rapid assessment of treatment responses. In a prospective cohort of critically ill patients on mechanical ventilation, a higher bacterial burden was associated with fewer ventilator‐free days. Metabolites offer a cost‐effective approach for diagnosing lung diseases, with lower immune toxicity and more controllable pharmacokinetics compared to other treatments. In the context of lung transplantation, the lung microbiome may serve as an indicator of chronic rejection or mortality. Similarly, in critically ill patients, an elevated bacterial DNA load is a reliable predictor of poor outcomes, suggesting that pulmonary microbiota profiles could indicate prognosis in the ICU. Additionally, the transient colonization patterns of the lung microbiome may allow for the monitoring of dynamic changes in lung cancer. Therapeutically targeting the lung microbiome is also relatively straightforward compared to other factors contributing to cancer heterogeneity, offering potential clinical advantages.

## Author Contributions

**Sana Arooj:** conceptualization, writing – original draft, writing – review and editing. **Akmal Zubair:** investigation, writing – review and editing, visualization, validation; methodology, project administration, software, supervision, resources, writing – original draft. **Syeda Zaira Batool:** methodology, visualization, writing – review and editing. **Granaz Niaz:** methodology, validation, conceptualization, funding acquisition. **Muhammad Ali:** investigation, funding acquisition, validation, visualization. **Yasir Waheed:** funding acquisition, writing – review and editing, project administration. **Eman Ramadan Elsharkawy:** conceptualization, methodology, visualization. **Naila Afghan:** formal analysis, project administration, software, methodology, validation.

## Ethics Statement

The authors have nothing to report.

## Conflicts of Interest

The authors declare no conflicts of interest.

## Data Availability

Data sharing not applicable to this article as no datasets were generated or analysed during the current study.
